# Lipid Droplets Formation Represents an Integral Component of Endothelial Inflammation Induced by LPS

**DOI:** 10.3390/cells10061403

**Published:** 2021-06-06

**Authors:** Krzysztof Czamara, Marta Stojak, Marta Z. Pacia, Alicja Zieba, Malgorzata Baranska, Stefan Chlopicki, Agnieszka Kaczor

**Affiliations:** 1 Jagiellonian Centre of Experimental Therapeutics (JCET), Jagiellonian University, Bobrzynskiego 14, 30-348 Krakow, Poland; krzysztof.czamara@uj.edu.pl (K.C.); marta.stojak@jcet.eu (M.S.); marta.pacia@jcet.eu (M.Z.P.); m.baranska@uj.edu.pl (M.B.); stefan.chlopicki@jcet.eu (S.C.); 2 Faculty of Chemistry, Jagiellonian University, Gronostajowa 2, 30-387 Krakow, Poland; ala.slaby@gmail.com; 3 Pharmacology Department, Jagiellonian University Medical College, Grzegorzecka 16, 31-531 Krakow, Poland

**Keywords:** lipid droplets, Raman imaging, atomic force microscopy, endothelium, inflammation, lipopolysaccharides

## Abstract

Endothelial inflammation is the hallmark of vascular pathology often proceeding with cardiovascular diseases. Here, we adopted a multiparameter approach combining various imaging techniques at the nano- and microscale (Raman, AFM and fluorescence) to investigate endothelial inflammation in response to lipopolysaccharides (LPS) in vitro in human microvascular endothelial cells (HMEC-1) with a focus on lipid droplets (LDs) formation. Our results show that LPS-induced LDs in HMEC-1 have a composition depending on LPS-incubation time and their formation requires the presence of serum. Robust endothelial inflammation induced by LPS was linked to LDs composed of highly unsaturated lipids, as well as prostacyclin release. LPS-induced LDs were spatially associated with nanostructural changes in the cell membrane architecture. In summary, LDs formation represents an integral component of endothelial inflammation induced by LPS.

## 1. Introduction

Endothelial inflammation is a well-known pathophysiological element of vascular diseases such as atherosclerosis or hypertension. Endothelial inflammation takes part in the early stages of pathology progression and involves the secretion of various proinflammatory and pro-thrombotic mediators, as well as the impaired function of vasoprotective endothelial mediators. Endothelial cells (ECs) play a key role in the immune response and inflammation [1,2] and mediate the migration of leukocytes through the endothelial barrier mediated by selectins, and adhesive proteins of the immunoglobulin family, e.g., ICAM-1 and VCAM-1 [1]. Endothelial inflammation may be triggered by pro-inflammatory factors, for example, tumor necrosis factor alpha (TNF-α) or lipopolysaccharides (LPS), after binding to specific membrane receptors, tumor necrosis factor receptor 1 (TNF-R1), and toll-like receptor 4 (TLR-4), respectively. Obviously, the number of possible mechanisms and pathways involved in endothelial inflammation by far exceed these two examples [2].

LPS is a major component of the cell wall of most Gram-negative bacteria and is comprised of a polysaccharide chain, determining the immunogenicity, and a lipid moiety called lipid A, which is also the bioactive component of LPS [3]. In bacterial infection, the cells react to LPS, i.e., the building blocks of bacteria cell walls triggering an inflammatory immune response that can lead to sepsis that is featured by profound ECs dysfunction. LPS induced ECs response is mediated by the soluble form of CD14 (sCD14) released by various cells into serum and plasma [4,5]. In contrast to monocytes and macrophages, ECs do not express CD14. Alternatively, the LPS-induced endothelial response is mediated by TLR-4 and the subsequent activation of nuclear protein complex NF-κB responsible for the transcription of relevant genes [6] including cyclooxygenase-2 resulting in increased synthesis of eicosanoids, including PGI_2_ [7].

Lipid droplets (LDs) are cellular organelles mainly composed of triacylglycerols and cholesteryl esters intrinsically related to normal cellular and organismal energy metabolism [8]. For years, they were considered only as lipid storage products, but recent studies have shown their active role in many cellular processes [9]. The main functions of LDs are the accumulation of lipids, protein binding and inactivation, intracellular transport of fats, e.g., to the mitochondria, and intracellular signaling. The participation of LDs in pathological processes, including inflammation and cancer, manifested by their increased biosynthesis in cells, has also been confirmed [10].

Taking advantage of 3D Raman microscopy, we have recently shown [11,12] that pro-inflammatory cytokine TNF-α evokes the formation of numerous highly unsaturated LDs in HMEC-1 cells that we ascribed as a hallmark of endothelial inflammation [11]. AFM–based measurements of ECs stimulated with proinflammatory factors, mainly with TNF-α [13,14,15,16,17,18,19] also demonstrated increased cell stiffness and decreased NO secretion and nanostructural changes in the cell membrane [17,18,19], but their pathophysiological relevance to endothelial inflammation was not clear.

In this work, applying a multiparameter approach combining Raman, AFM, and fluorescence microscopy, we studied LPS-triggered endothelial inflammation. Our aim was to characterize the LDs’ chemical content in response to LPS and their relevance to the inflammation and PGI_2_ production in the presence or absence of serum, and test whether LPS-induced endothelial inflammation is related to changes in the morphology of the cell membrane. We confirmed that the formation of LDs in the endothelium was directly related to endothelial inflammation and triggered changes in the nanostructure of the cell membrane of ECs.

## 2. Materials and Methods

### 2.1. Cell Culture 

To study inflammation in the endothelium, the immortalized cells line HMEC-1 (human dermal microvascular endothelial cells; American Type Culture Collection company, USA) [20] were activated with LPS. The cells for Raman measurements were prepared as previously described [11]. After 24 h of incubation, the cells were washed twice with phosphate-buffered saline (PBS, Gibco Life Technologies, Warszawa, Poland) and exposed to lipopolysaccharides from *Escherichia coli* O111:B4 (LPS, Sigma Aldrich, Poznań, Poland) in concentrations of 0.01, 0.1, 1.0 and 10 μg·mL^−1^ dissolved in a fresh medium for 24 h. To investigate the influence of incubation time, an additional sample of HMEC-1 held for 48 h in the LPS concentration of 10 μg·mL^−1^ was prepared. Untreated HMEC-1 maintained in the medium for 24 and 48 h, respectively, were used as a control. Additionally, the influence of FBS on lipid droplet formation was studied for HMEC-1 in the experiment with no supplementation of FBS in the MCDB131 (otherwise the protocol as above). In this case, the cells were starved for 24 and 48 h. After stimulation, the cells were rinsed twice with PBS and fixed with glutaraldehyde 2.5% solution (Sigma Aldrich, Poland) in PBS for 4 min. The samples were stored in PBS at 4°C until Raman measurements.

### 2.2. Raman and AFM Microscopy

Raman imaging and AFM analysis were performed using a WITec Confocal Raman Imaging system (WITec alpha300, Ulm, Germany) equipped with a 532 nm laser, a UHTS 300 spectrograph (600 grooves·mm^−1^ grating), a CCD detector (DU401A-BV-352, Andor, UK) and a 63× water immersion objective (NA = 1.0, Zeiss Fluor, Germany). Raman spectra were acquired with a sampling density of 0.3 µm in *x*/*y* directions and a 0.5 s exposure time per spectrum using the maximum laser power at the sample (*ca.* 30 mW) was selected. For each group (control, LPS in 0.01, 0.1, 1.0, and 10 μg·mL^−1^ concentrations) areas of at least 5 cells were measured. Raman imaging of HMEC-1 with LPS in the concentration of 10 μg·mL^−1^ was performed in four independent biological replicants. Raman 3D profiling experiments were carried out by repeatedly measuring the area of the entire cell changing the focal distance (5 layers in 1 µm step in the *z*-axis). The distribution images collected at different depths present the relative intensity of a studied component in the cells. 

The Raman spectra of lipid standards were measured on CaF_2_ slides with the application of the 100× air objective (NA = 0.90, Olympus MPLAN, Japan) with the analogous WITec system. For each spectrum, 100 scans were collected and an integration time of 0.5 s using maximum *ca*. 20 mW laser power at the sample position.

After the Raman measurements, AFM was used to investigate the topographic and structural changes on the endothelial cell surface induced by LPS stimulation. AFM measurements were performed in the tapping (AC) mode with the force modulation probes (k = 0.2 N/m, WITec, Ulm, Germany) using the 20× immersive objective (NA = 0.5, Olympus, Japan). 

### 2.3. Expression of ICAM-1 

The HMEC-1 were seeded onto 96-well plates at a concentration of 3·10^4^ cells per well, left to grow for 24 h to the 100% confluence, and then incubated with LPS in various concentrations (0.01, 0.1, 1.0, 10, 100 µg·mL^−1^). After 24 h, the cells were stained with mouse anti-human CD54-PE (BD Pharmingen, San Jose, CA, USA), according to the manufacturer’s protocol, and with Hoechst 33,342 (Life Technologies, Warszawa, Poland) for 10 min. The expression of surface ICAM-1 was observed by fluorescence microscopy (a ScanR screening system) in randomly selected 8 visual fields for each well and was analyzed using Columbus 2.4.2 Software (Perkin Elmer, Waltham, MA, USA). The experiments were performed in triplicate and repeated three times.

### 2.4. MTS Assay 

The HMEC-1 were plated onto 96-well tissue culture plates at a concentration of 3 × 10^4^ cells per well and left to grow to the 100% confluence. Then, the cells were incubated with LPS at various concentrations (0.01, 0.1, 1, 10, 100 µg·mL^−1^). After 24 h incubation, the viability of the cells was measured using MTS tetrazolium substrate (CellTiter 96 AQueous Non-Radioactive Cell Proliferation Assay, Promega) according to the manufacturer’s protocol. The absorbance was measured at 490 nm using a spectrophotometer (Synergy 4, BioTek, VT, USA). All the experiments were repeated three times with at least triplicates for each concentration.

### 2.5. BODIPY Staining of Lipid Droplets 

Cytosolic lipid droplets were stained using BODIPY 493/503 (4,4-Difluoro-1,3,5,7,8-Pentamethyl-4-Bora-3a,4a-Diaza-s-Indacene, Molecular Probes, Eugene, OR, USA) according to the manufacturer’s protocol. Briefly, HMEC-1 were plated onto 96-well tissue culture plates at a concentration of 3 × 10^4^ cells per well and incubated with LPS at various concentrations for 24 h. Then, cells were washed two times with PBS (pH 7.4, Gibco Life Technologies, Poland) and fixed with 4% paraformaldehyde (PFA) for 10 min at room temperature and finally stained with BODIPY for 30 min. HMEC-1 were then washed with PBS and stained with CellMask Deep Red (Molecular Probes, USA) and Hoechst 33,342 (Molecular Probes, USA) for 30 min at room temperature and analyzed at 20× magnification objective on CQ1 Confocal Quantitative Image Cytometer (Yokogawa, Tokyo, Japan). All experiments were repeated two times with ten replications for each concentration.

### 2.6. Assessment of LPS Induced PGI_2_ Release in HMEC-1

Measurements of PGI_2_ production in the cell culture media were quantified based on the formation of its stable metabolite, 6-keto PGF_1α_. The assay for 6-keto-PGF_1α_ in the effluent from HMEC-1 cultured in medium with/without FBS and LPS (10 µg·mL^−1^) for 24 h and 48 h was quantified using a commercially available ELISA kit (Cayman Chemical, Ann Arbor, MI, USA).

### 2.7. Data Analysis and Processing

The data matrices of Raman spectra were baseline corrected using autopolynomial of degree 3 and submitted to a cosmic ray removal procedure using the WITec Project Plus software. Cluster analysis (KMCA) was performed to group data into classes and extract the average spectra reflecting the major organelles i.e., LDs with the *k*-means method using the Manhattan distance (WITec Project Plus). The analysis of variance (the ANOVA model with the Tukey’s test) was performed in the OriginPro 9.1 software to quantitatively characterize the differences in biochemical content in all pairwise comparisons for each group (control and incubated with LPS in various concentrations).

## 3. Results

### 3.1. LPS Induces Formation of Lipid Droplets

**Figure 1:**
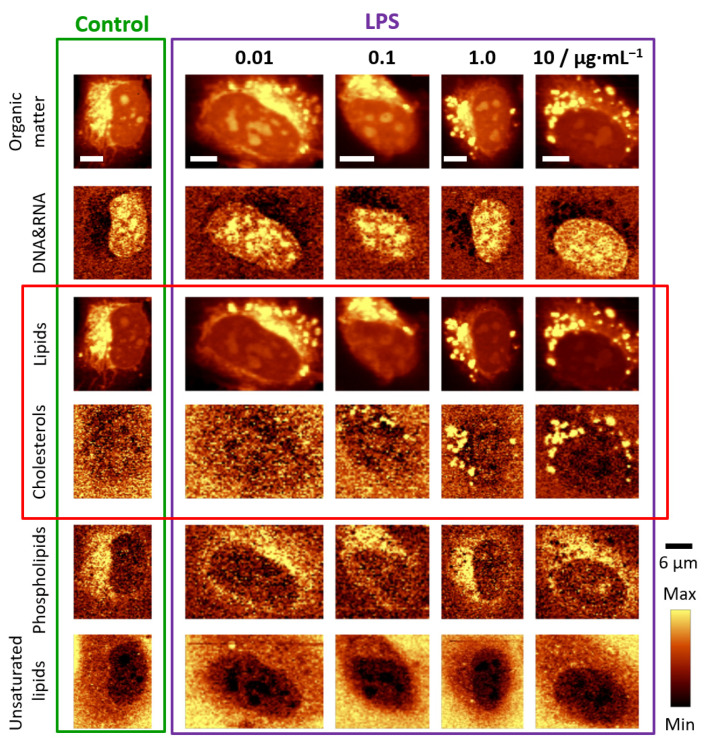
Representative Raman images of endothelial cells: control and incubated with LPS for 24 h. Raman images of distribution in HMEC-1 obtained by integration in the following spectral regions: 3030–2830 cm^−1^ (organic matter), 810–760 cm^−1^ (DNA & RNA), 2900−2830 cm^−1^ (lipids), 715–695 cm^−1^ (cholesterols), 733–713 cm^−1^ (phospholipids) and 3030–3000 cm^−1^ (unsaturated lipids). Scale bars equal 6 μm.

**Figure 2:**
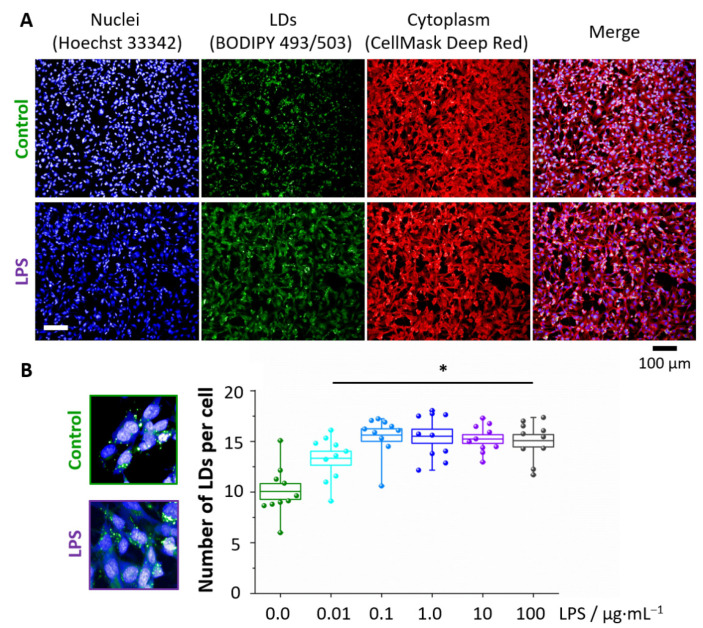
Formation of LDs in LPS-stimulated endothelial cells. Representative fluorescence images (**A**) of control and LPS-treated HMEC-1 (10 μg·mL^−1^, 24 h) showing the distribution of nuclei (blue, Hoechst 33342), lipid droplets (green, BODIPY 493/503) and cytoplasm (red, Cell Mask Deep Red) that enabled for calculations of the number of LDs per cell (**B**). Values given as mean ± SEM are shown in box plots: mean (horizontal line), SEM (box), minimal and maximal values (whiskers). * *p* < 0.05.

To investigate LPS-triggered alterations in the chemical composition of HMEC-1, the averaged Raman spectra of the cells were extracted and subsequently averaged over spectra for all single cells measured in a given group. The obtained Raman spectra for control and LPS-stimulated cells at concentrations of 0.01, 0.1, 1.0, and 10 μg·mL^−1^ were compared and the analysis of integral intensities of characteristic bands were performed (Figures S1 and S2, SI). The spectral profiles of cells in all studied groups were very similar and only subtle differences arising from the increased cholesterol level were observed in the Raman spectra of cells treated with LPS at the concentration of 10 μg·mL^−1^. The abovementioned findings, i.e., the observation that tremendous chemical alterations in subcellular organelles were not manifested in the averaged Raman spectra of cells, highlighted the necessity of using high spatial resolution imaging. The effect of increasing concentrations of LPS on the chemical composition and distribution of organelles inside ECs was studied using Raman and fluorescence imaging, and the results are presented below (Figure 1 and Figure 2).

Two-dimensional Raman images were based on the analysis of characteristic bands, in particular at 785 cm^−1^ due to the ring breathing modes of nitrogen bases in the DNA&RNA, at 724 cm^−1^ due to the symmetric stretching vibrations of the choline N^+^(CH_3_)_3_ groups of phospholipids, and at 704 cm^−1^ due to the in-plane ring deformations of cholesterols. Additionally, the Raman signal in the 2900−2830 cm^−1^ spectral range associated with the C–H stretching vibrations, originating mostly from lipids, as well as the band in the 3030–3000 cm^−1^ range due to lipids containing double bonds in their hydrocarbon chains, were considered [21]. In addition, Raman images presenting the distribution of all organic compounds are shown. The obtained images enabled us to draw the following conclusions. HMEC-1 incubated with LPS form numerous LDs which composition was dominated by cholesterols (Figure 1, marked with a red rectangle). The onset of LDs formation was visible for the LPS concentration of 1.0 μg·mL^−1^ but starting from the 10 μg·mL^−1^ concentration this effect was evident for the whole measured population of cells. Unlike LDs observed after stimulation with TNF-α [11], LDs triggered by 24 h incubation with LPS did not exhibit a high degree of lipid unsaturation. In principle, there was no change in lipid unsaturation compared to the unsaturation of LDs in the control (scarce amount, *vide infra*). In addition, there were no significant alterations in the distribution of other biomolecules in the studied cells. 

Fluorescence microscopy was used to investigate the concentration-dependence of the LDs formation in response to LPS (Figure 2). In Figure 2A the BODIPY staining visualizes the intracellular distribution of LDs, showing that for all concentrations the statistically significant increase in the number of LDs per cell after LPS stimulation occurred in comparison to the control. However, starting from the LPS concentration of 0.1 μg·mL^−1^, the number of LDs was constant, most probably due to droplets’ aggregation at higher LPS concentrations.

### 3.2. LPS Induces Changes in the Topography of the HMEC-1 Cell Membrane

**Figure 3:**
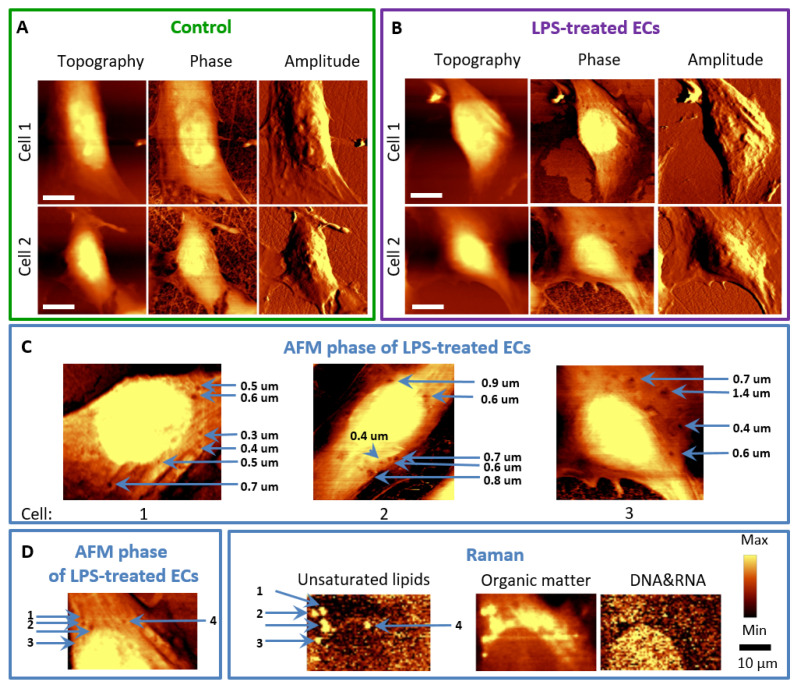
AFM and Raman imaging of endothelial cells incubated with LPS. Representative images of the topography, phase, and amplitude of control HMEC-1 cells (**A**) and stimulated with LPS at a concentration of 10 μg·mL^−1^ for 24 h (**B**). The arrows (**C**) indicate the locations of concaves in the phase images of LPS-treated cells. Phase and topography AFM image as well as Raman images showing co-localized changes in the cell membrane and inside of the cell (**D**).

LPS, as a membrane-interacting molecule, may affect its properties. The effect of LPS on the cell membrane architecture of HMEC-1 was investigated using parallel AFM-Raman imaging. The biggest difference between the control and LPS-stimulated ECs was the appearance of concaves on the surface of LPS-stimulated cells (Figure 3).

The control cells were spindle-shaped, while after incubation with LPS, the cells changed into a more spherical shape (Figure 3A,B). Moreover, the inhibition in the formation of filopodia (substrate exploring parts of the lamellipodium) [22] could be observed for LPS-treated cells in comparison to control cells, but this effect will require further investigation Similar morphological changes were previously reported after TNF-α in HMEC-1 that lost their cobblestone-pattern structure, and contracted, as a consequence of cytoskeleton reorganization [15,16,18]. It is known that TNF-α stimulation caused diversification in F-actin location and structure and longer TNF-α exposure (6–24 h) resulted in F-actin depolymerization, observed as a shortening of fibers and their irregular orientation [15,16,18]. The role of small GTPases: RhoA and RhoG and filamin were suggested to regulate the abovementioned cytoskeletal rearrangements, in particular, RhoA and RhoG inducing cytoskeleton rearrangement, necessary for both paracellular (the opening of junctions) and transcellular pathways (directly through ECs) [2,23].

For LPS, changes in the ECs’ membrane architecture were also observed (Figure 3C). After incubation of HMEC-1 with LPS, small concaves reaching a diameter of several dozen nanometres in the cell membrane emerged (Figure 3C, marked with blue arrows). AFM phase images showed that in the same places as the concaves on the topography images, viscoelastic changes at these sites in the cell membrane were observed. Their size was different, and the diameter did not exceed one micrometre, there was, however, an evident co-localization of LDs observed in Raman images and concaves appearing in the topography and phase images (Figure 3D). It is hypothesized that LDs induce a collapse of the membrane and changes in the viscoelastic properties in their proximity, which was previously observed for HUVEC [24]. This effect explains also the increased permeability of the endothelial membrane upon inflammation [25,26,27,28].

### 3.3. Chemical Composition of LDs Triggered by LPS Depends on Incubation Time

**Figure 4:**
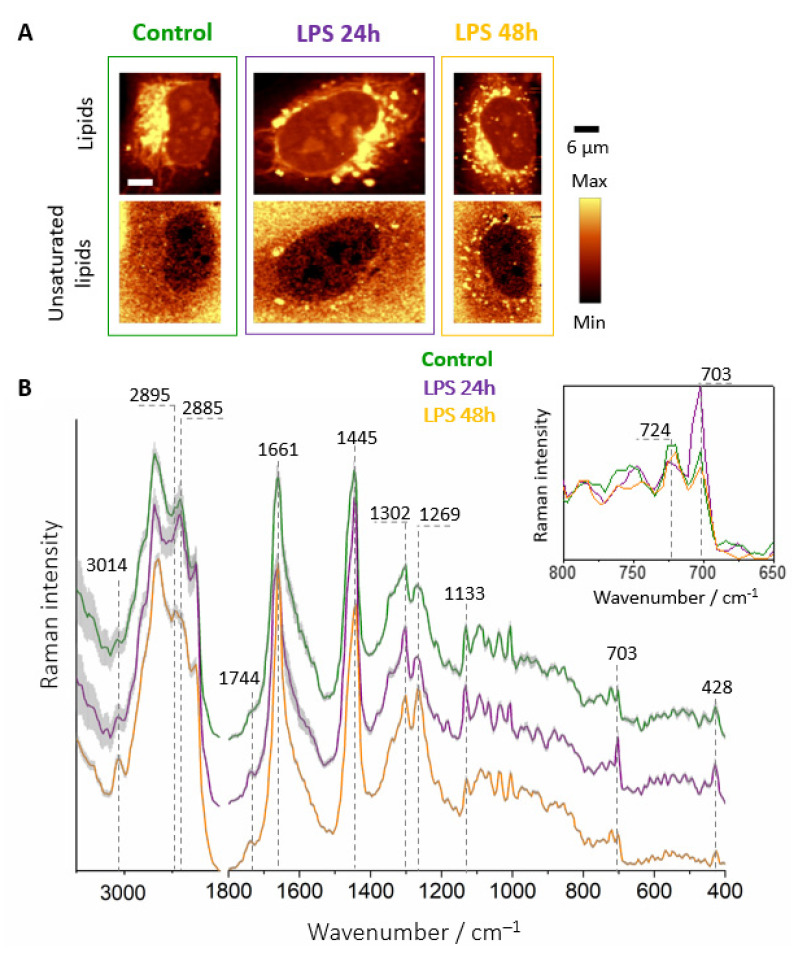
Raman imaging of LDs in endothelial cells. Representative Raman images of control and incubated with LPS (10 μg·mL^−1^) HMEC-1 for 24 and 48 h (**A**) in FBS supplemented media and Raman spectra of LDs (**B**) extracted from control and LPS-stimulated cells for 24 h (violet) and 48 h (gold) averaged over all measured cells. The insert shows changes in the intensity of the bands at 724 and 704 cm^−1^. Spectra were normalized in the 1500–400 cm^−1^ spectral range and were presented with the standard error on each data point (grey area), and have been shifted vertically for clarity.

The chemical composition of LDs formed within HMEC-1 cells treated with LPS was determined using a KMCA analysis of Raman data. This approach enabled us to extract, classify, and compare spectra of LDs from control and LPS-stimulated cells [11,29]. The Raman spectra obtained for the LDs classes were averaged and normalized, and the spectral profiles with the standard deviation at each point (gray area) are shown in Figure 4.

Although the number of LDs in the 24 h LPS-treated cells was significantly higher than for the control (Figure 4A), their chemical composition was quite similar as demonstrated by their Raman signatures (Figure 4B). They exhibited a typical spectral profile of lipids with characteristic bands at 2885, 1445, and 1302 cm^−1^ corresponding to the hydrocarbon chain vibrations. Moreover, bands due to unsaturated lipids at 3014, 1661, and 1269 cm^−1^ arising from the vibrations associated with the C=C group [21] were observed both in the control and LPS-stimulated cells. The major differences in the spectra were revealed as the increased intensity of the bands at 1744, 704, and 428 cm^−1^, originating from the C=O stretching modes (1744 cm^−1^) and ring deformations of the cholesterol esters (704 and 428 cm^−1^), respectively, in the spectra of 24 h LPS-treated cells. 3D imaging (Figure S3, SI) showed the coexistence of two types of LDs, saturated LDs that dominated among observed entities (green areas in KMCA images and respective Raman spectra) and much less numerous LDs of highly unsaturated chemical character. In their Raman spectra, intense bands of unsaturation could be observed at 3014, 1661 and 1269 cm^−1^. In addition, intense bands derived from cholesterol esters were visible at 1744, 704 and 428 cm^−1^ that co-localized with unsaturation.

The Raman images obtained after prolonged incubation time with LPS (48 h, Figure 4A) exhibited numerous LDs in the cytoplasm. Significantly, their spectral profile was characterized by intensive bands at 1269, 1661, and 3014 cm^−1^ arising from unsaturated hydrocarbon chain vibrations. Additionally, bands originating from cholesterols at 703 and 428 cm^−1^ showed decreased intensity in comparison to the Raman spectrum of cells after 24 h incubation. Moreover, the Raman profile of HMEC-1 after 48 h of treatment was strikingly similar to the Raman spectrum of the same cell line stimulated with TNF-α, especially in terms of the degree of unsaturation [11]. This finding highlights the common feature of inflamed endothelial cells manifested by the formation of highly unsaturated LDs. Regardless of the proinflammatory agent, after appropriate incubation time, the observed composition of lipids in LDs is very similar and independent of the trigger. 

### 3.4. Absence of FBS Prevents Formation of Lipid Droplets in Response to LPS 

**Figure 5:**
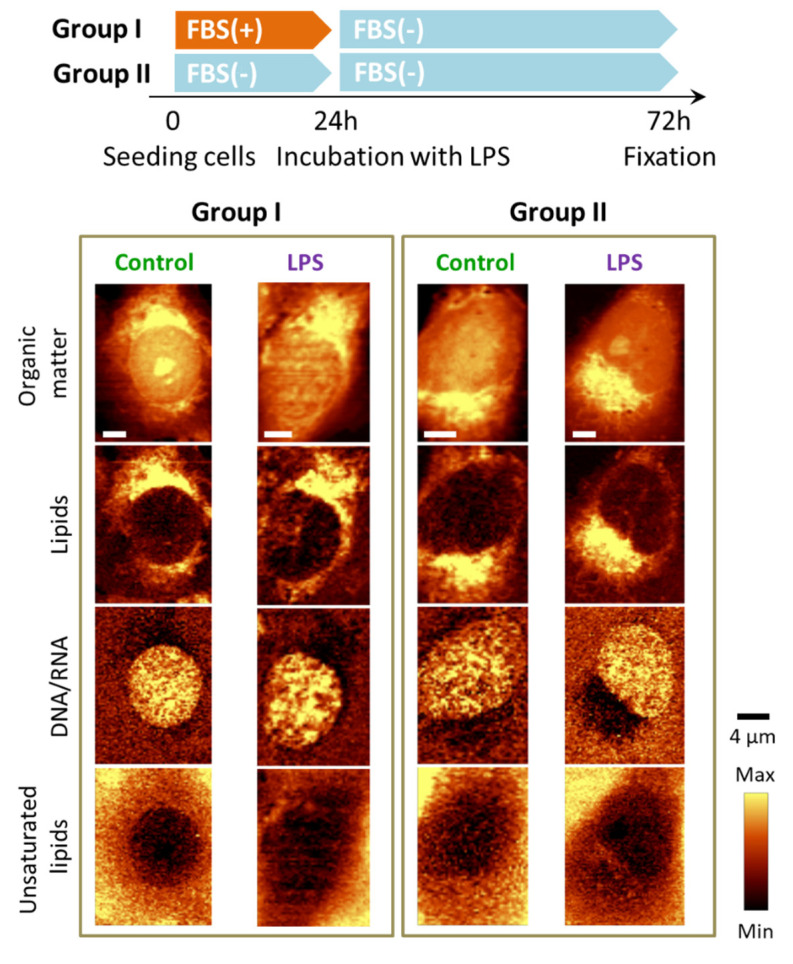
LPS-induced LDs formation in endothelial cells in the absence of FBS. Representative Raman images of the distribution of HMEC-1 incubated without FBS supplementation in various seeding conditions (absence/presence of LPS) obtained by integration in the following spectral regions: 3030–2830 cm^−1^ (all organic matter), 2900−2830 cm^−1^ (lipids), 810−760 cm^−1^ (DNA & RNA) and 3030–3000 cm^−1^ (unsaturated lipids).

The activation of two proteins i.e., LPS-binding protein (LBP) and CD14 [4] is required to bind the LPS molecule to TLR-4. ECs either do not express CD14 at all [4,30] or only at low concentrations (for example on the HUVEC surface) [31]. Both LBP and CD14 are, however, found in the FBS serum supplemented medium. It has been shown that ECs respond to low concentrations of LPS only in the presence of serum containing sCD14 [25]. To confirm that the mechanism of LPS activation in inflamed endothelium manifested by subsequent LDs formation was linked with FBS, we performed an experiment in which HMEC-1 were treated with LPS in a medium without FBS supplementation for 24 and 48 h. The results of the Raman imaging are presented below (Figure 5).

The Raman images show that ECs maintained in FBS-deficient medium did not form LDs in response to LPS most probably due to lack of FBS proteins (LBP and CD14) required to form complex with LPS enabling to bind to TLR-4 and trigger subsequent cellular events of the inflammatory process. To directly demonstrate that LPS in the presence of FMS did not result in apoptosis, a viability test (Figure S4, Supplementary Materials) was performed. The viability test results showed that LPS in a wide range of concentrations did not cause any significant damage to HMEC-1 (Figure S4, SI), as found previously for human pulmonary microvascular endothelial cells (HPMEC) [32]. 

**Figure 6:**
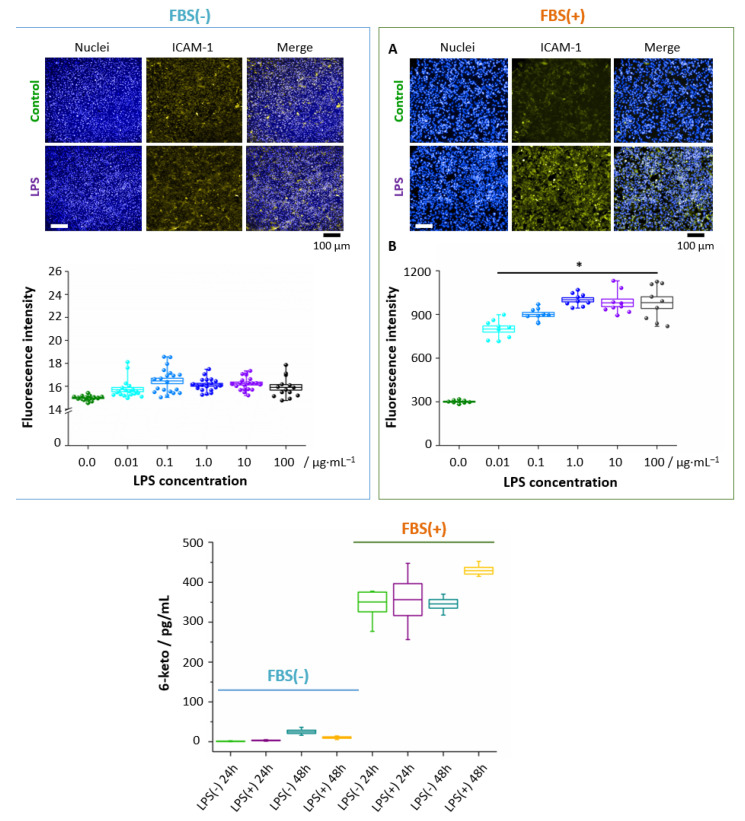
LPS-induced ICAM-1 expression and PGI_2_ production in endothelial cells in the presence and absence of FBS. The staining of surface ICAM-1 molecules was performed for HMEC-1 after 24 h treatment with LPS *E. Coli* in different concentrations. Representative fluorescence images (**A**) showed the overexpression of ICAM-1 (fluorescence intensity of the fluorophore (R-phycoerythrin) fused to the ICAM-1 antibody, yellow areas, nuclei – blue areas) for LPS-treated cells (10 μg·mL^−1^) in comparison to control cells, combined with the analysis of fluorescence intensity (**B**). The concentration of 6-keto PGF1α in effluent from HMEC-1 from control and LPS-stimulated (10 μg·mL^−1^) HMEC-1 with/without FBS supplementation (**C**). Values given as mean ± SEM are shown in box plots: mean (horizontal line), SEM (box), minimal and maximal values (whiskers). * *p* < 0.05.

To determine the pro-inflammatory impact of LPS, immunohistochemical staining for membrane ICAM-1, i.e., a cell surface glycoprotein typically present in the membrane of ECs and cells of the immune system, overexpressed due to pro-inflammatory agents, was performed [33]. Our results demonstrate (Figure 6A,B) that LPS, in the presence of FBS, even at a concentration of 0.01 μg·mL^−1^, causes a statistically significant increase in yellow fluorescence intensity (indicating the ICAM-1 presence), which gradually increases for higher concentrations and reaches a *plateau* at the 1.0 μg·mL^−1^ concentration of LPS.

This finding provides the evidence of LPS-induced inflammation in HMEC-1. Importantly, in the absence of FBS, the inflammation was not observed. To highlight the proinflammatory effect of LDs and direct relation with activation of the arachidonic acid pathway, 6-keto PGF_1α_ (6-keto PGF_1α_, a non-enzymatic hydrolysis product of prostacyclin) was investigated for ECs incubated with LPS in the 10 μg·mL^−1^ concentration (Figure 6C). A marked elevation of 6-keto PGF_1α_ level after 48 h incubation with LPS shows directly that the PLA2/COX/PGIS pathway involving arachidonic acid was activated in the HMEC-1 upon prolonged incubation with LPS, in agreement with the increase of the lipid unsaturation level with time (Figure 4; arachidonic acid is polyunsaturated 20:4(ω-6)). 

Therefore, these findings explicitly show the essential role of FBS in inflammation and activation of the arachidonic acid pathway yielding a possible explanation for a change in the chemical character of formed LDs toward unsaturated lipids. Additionally, our results directly demonstrate that the formation of LDs and inflammation/arachidonic acid pathway activation are linked.

## 4. Discussion

The formation of unsaturated lipid droplets in the endothelial cells was previously indicated as a hallmark of inflammation [11,12]. Our new results show that depending on the trigger and/or progression of inflammation, it may lead to the formation of droplets varying in the chemical composition. For LPS-triggered inflammation, the ECs responded initially through the formation of LDs rich in saturated lipids and cholesterol esters. Nevertheless, ‘late stages’ of endothelial inflammation (48 h LPS, 24 h TNF-α) were marked by the presence of LDs of an unsaturated character, which was related to the activation of the arachidonic acid pathway and inflammation, compatible with the increased 6-keto PGF_1α_ and ICAM-1 levels, respectively.. Importantly, Raman signatures of ‘late stage’ endothelial inflammation were strikingly similar for LPS and TNF-α, showing that in the advanced stage Raman markers of inflammation (i.e., unsaturated LDs) were trigger-independent for comparison of LPS and TNF-α.

Apart from LDs formation inside the cells, endothelial inflammation is related to various changes in the cell membrane. In particular, F-actin depolymerization is directly related to increased endothelial permeability and has enormous consequences in the context of endothelial dysfunction [28]. As long as the endothelium is a continuous monolayer, it controls the passage of molecules from the bloodstream into the surrounding tissues, but changes in the cytoskeleton under the influence of proinflammatory factors, such as LPS, cause disruption of ECs’ monolayer integrity and increased endothelial permeability [25,26,27,28] that also involves the formation of gaps in endothelial junctions, leading to plasma leakage, a hallmark of severe sepsis and septic shock [25,34,35]. As was reported in the literature, the movement of ^14^C-bovine serum albumin across bovine pulmonary artery endothelial cell monolayers significantly increased after 6 h exposure to LPS (in the concentration at 10 µg·mL^−1^), accompanied by actin reorganization [28]. Additionally, the HUVEC monolayers exposed to LPS and TNF-α showed increased permeability, determined by the transport of albumin and dextran through the monolayer, by about 2.5-fold in comparison to the control [26]. Our results showed that nanostructured changes in the cell membrane architecture, observed after 24 h LPS incubation, were related spatially with the areas inside cells where LDs were formed. Although it is not clear if changes in the cell membrane, i.e., concaves of different viscoelastic properties (observed in AFM images) preceded the formation of LDs (manifested in Raman images) or *vice versa*, it is clear that bulk properties of the cells changed upon inflammation. Finally, we determined that nanoscale changes observed in the membrane of inflamed endothelial cells (concaves of different physical properties) and microscale changes inside the cells (formation of lipid droplets) were interrelated phenomena that could also be linked mechanistically.

**Figure 7:**
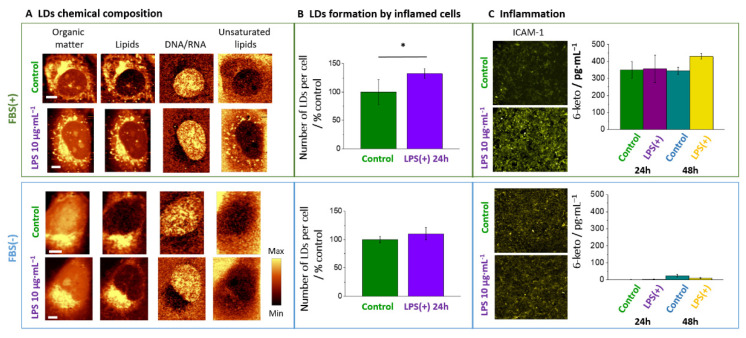
LPS-induced response of endothelial cells in the presence and absence of FBS. In the presence but not in the absence of FBS, LPS-induced LD formation rich in unsaturated lipids (**A**,**B**) was directly related to inflammation manifested as increased ICAM-1 expression and elevated 6-keto PGF_1α_ level (**C**). * *p* < 0.01.

To conclude (Figure 7), our work showed that LDs formation was an inherent component of endothelial inflammatory response and was absent in the absence of serum, which eliminated LPS-induced endothelial inflammatory response. A biochemical content of LDs formed in response to LPS was dependent on LPS concentration and incubation time with more unsaturated character linked with ‘late stage’ of inflammation associated with the activation of the eicosanoids release and significant changes in the nanostructure of the endothelial cell membrane.

## Supplementary Materials

The following are available online at https://www.mdpi.com/article/10.3390/cells10061403/s1, Figure S1: Comparison of Raman spectra of control and LPS-stimulated HMEC-1. Raman spectra from control (green) and LPS-stimulated for 24 h in concentrations of 0.01, 0.1, 1.0 and 10.0 μg·mL^−1^ (cyan, blue, dark blue and violet, respectively) HMEC-1 averaged over all measured cells. Spectra were normalized in the 1500–400 cm^−1^ spectral range and have been shifted vertically for clarity. Figure S2: Chemical changes upon inflammation in endothelial cells induced by LPS. Integral intensity of marker bands at 1444, 1007, 785 and 704 cm^−1^ for control cells and LPS-stimulated cells showing alterations in the concentration of lipids, proteins, DNA&RNA and cholesterols within cellular components of HMEC-1 depending on LPS concentration. Figure S3: Confocal 3D imaging of a random LPS-stimulated HMEC-1 cell. Raman distribution image for representative 24 h LPS-stimulated (10 μg·mL^−1^) cell (A) obtained from layers every 1 μm step in the z-direction by integration in the spectral region of 3030–2830 cm^−1^ (all organic matter), 2900–2830 cm^−1^, 715–695 cm^−1^ (cholesterols), 3030–3000 cm^−1^ (unsaturated lipids) and 2900–2870 cm^−1^ (saturated lipids). Intensities of bands in all layers were normalized. The KMCA images (B) from the studied planes in which two classes were identified in colors: unsaturated LDs (red) and saturated LDS (green) with the corresponding averaged Raman spectra of classes (C). Spectra were normalized in the 1500–400 cm^−1^ spectral range and have been shifted vertically for clarity. Figure S4: Changes in cell viability in HMEC-1 treated with different concentrations of LPS. The MTS assay was performed for cells maintained in FBS-containing medium supplemented with LPS for 24 h. Values given as mean ± SEM are shown in box plots: mean (horizontal line), SEM (box), minimal and maximal values (whiskers).

## Abbreviations

6-keto PGF_1α_	6-keto prostaglandin F_1α_
AFM	Atomic Force Microscopy
AC	tapping mode in AFM
CD14 (sCD14)	(soluble) Cluster of Differentiation 14, serum protein
ECs	Endothelial cells
EA.hy926	immortalized hybrids, fusion of HUVEC with human lung carcinoma cell line A549
FBS	Fetal Bovine Serum
HCAEC	Human Coronary Artery Endothelial Cells
HMEC-1	Human dermal Microvascular Endothelial Cells transfected with pSVT vector, a pBR-322 based plasmid containing the coding region for Simian virus 40A
HPMEC	Human Pulmonary Microvascular Endothelial Cells
HUVEC	Human Umbilical Vascular Endothelial Cells
ICAM-1	Intercellular Adhesion Molecule 1
JAM-A	Junctional Adhesion Molecule-A
KMCA	*k*-means Cluster Analysis
LDs	Lipid Droplets
LPS	Lipopolysaccharides
NF-κB	Nuclear Factor Kappa-light-chain-enhancer of Activated B Cells
NO	Nitric Oxide
PGI_2_	Prostacyclin I_2_
SERS	Surface Enhanced Raman Spectroscopy
TLR-4	Toll-like Receptor 4
TNF-α	Tumor Necrosis Factor alpha
TNF-R1	Tumor Necrosis Factor Receptor 1
VCAM-1	Vascular Adhesion Molecule 1
