# Supplementary material for: Lipid Droplets Formation Represents an Integral Component of Endothelial Inflammation Induced by LPS
Source: Cells. 2021 Jun 6;10(6):1403. doi: 10.3390/cells10061403 (PMC8227392; doi:10.3390/cells10061403)
Supplement: Supplementary file 1 [file cells-10-01403-s001.zip › cells-1231529-supplementary.pdf]

cells

MDPI

Supplementary Information

Lipid droplets formation represents an integral component of endothelial inflammation induced by LPS

K. Czamara[1], M. Stojak[1], M. Z. Pacia[1], A. Zieba[2], M. Baranska[1,2], S. Chlopicki[1,3] and A. Kaczor[1,2,*]

[1]Jagiellonian Centre of Experimental Therapeutics (JCET), Jagiellonian University, Bobrzynskiego 14, 30-348 Krakow, Poland.
[2]Faculty of Chemistry, Jagiellonian University, Gronostajowa 2, 30-387 Krakow, Poland.
[3]Chair of Pharmacology, Jagiellonian University Medical College, Grzegorzecka 16, 31-531 Krakow, Poland.
*corresponding author e-mail: agnieszka.kaczor@uj.edu.pl

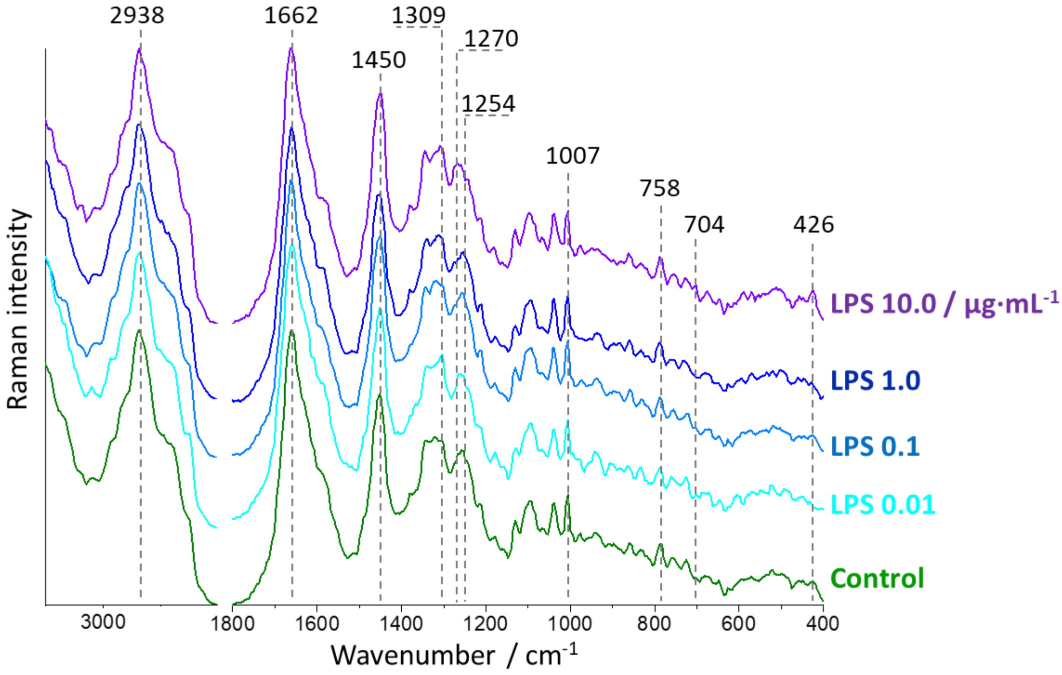

Figure S1. Comparison of Raman spectra of control and LPS-stimulated HMEC-1. Raman spectra from control (green) and LPS-stimulated for 24 hours in concentrations of 0.01, 0.1, 1.0 and 10.0 µg·mL^{-1} (cyan, blue, dark blue and violet, respectively) HMEC-1 averaged over all measured cells. Spectra were normalized in the 1500–400 cm^{-1} spectral range and have been shifted vertically for clarity.

The spectral profiles of cells in all studied groups are very similar. There are no significant changes in the average Raman spectra, especially at lower LPS concentrations, as compared to the Raman average spectrum of control cells. The spectral contour and position of the bands is typical for endothelial cells, and their origins have been already discussed in detail. The subtle differences are observed in the Raman spectra of cells treated with LPS at the concentration of 10 µg·mL^{-1}. In this case, the low-intensity bands at 704 and 426 cm^{-1} associated with the sterol ring deformations are observed, and their presence in the averaged spectra indicates a considerable increase in the concentration of cholesterols in the cells under the stimulation of LPS.

In order to better determine the chemical changes induced by LPS, the analysis of the level of biocomponents, i.e. proteins, lipids, or nucleic acids, was done, by determining the integral intensity for characteristic Raman signals (Fig. S2). Results for all stimuli indicate the insignificant, subtle changes in the concentrations of tested biocomponents. The calculated intensity values remain at the same level of the mean value for the control. However, small, but though statistically insignificant, increase in lipid content in cells for higher LPS concentrations are observed. At the LPS 10 μg·mL^{-1} concentration, this change can be approximated with 20% increase in the total cholesterols content, which is the most notable change.

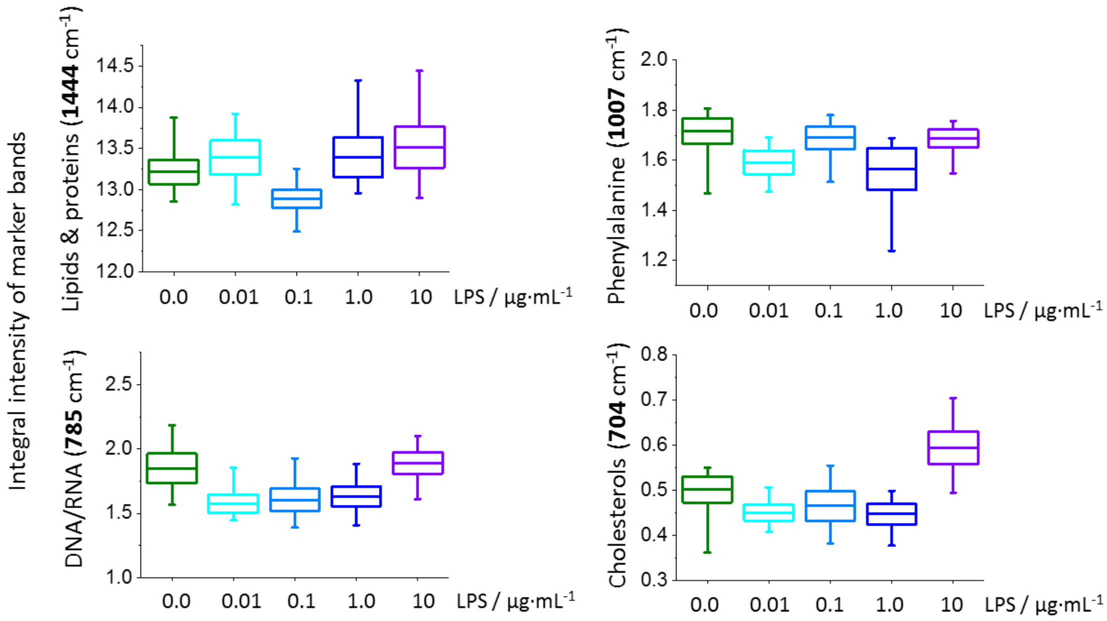

Figure S2. Chemical changes upon inflammation in endothelial cells induced by LPS. Integral intensity of marker bands at 1444, 1007, 785 and 704 cm^{-1} for control cells and LPS-stimulated cells showing alterations in the concentration of lipids, proteins, DNA&RNA and cholesterols within cellular components of HMEC-1 depending on LPS concentration.

To answer the question if the whole population of LDs in inflamed HMEC-1 is composed of saturated lipids, we performed a more detailed analysis of the spatial distribution and heterogeneity of formed LDs based on the Raman imaging and the KMCA analysis. The results of the Raman 3D profiling of the whole volume of a random HMEC-1 cell incubated with LPS for 24 h are shown below (Fig. S3).

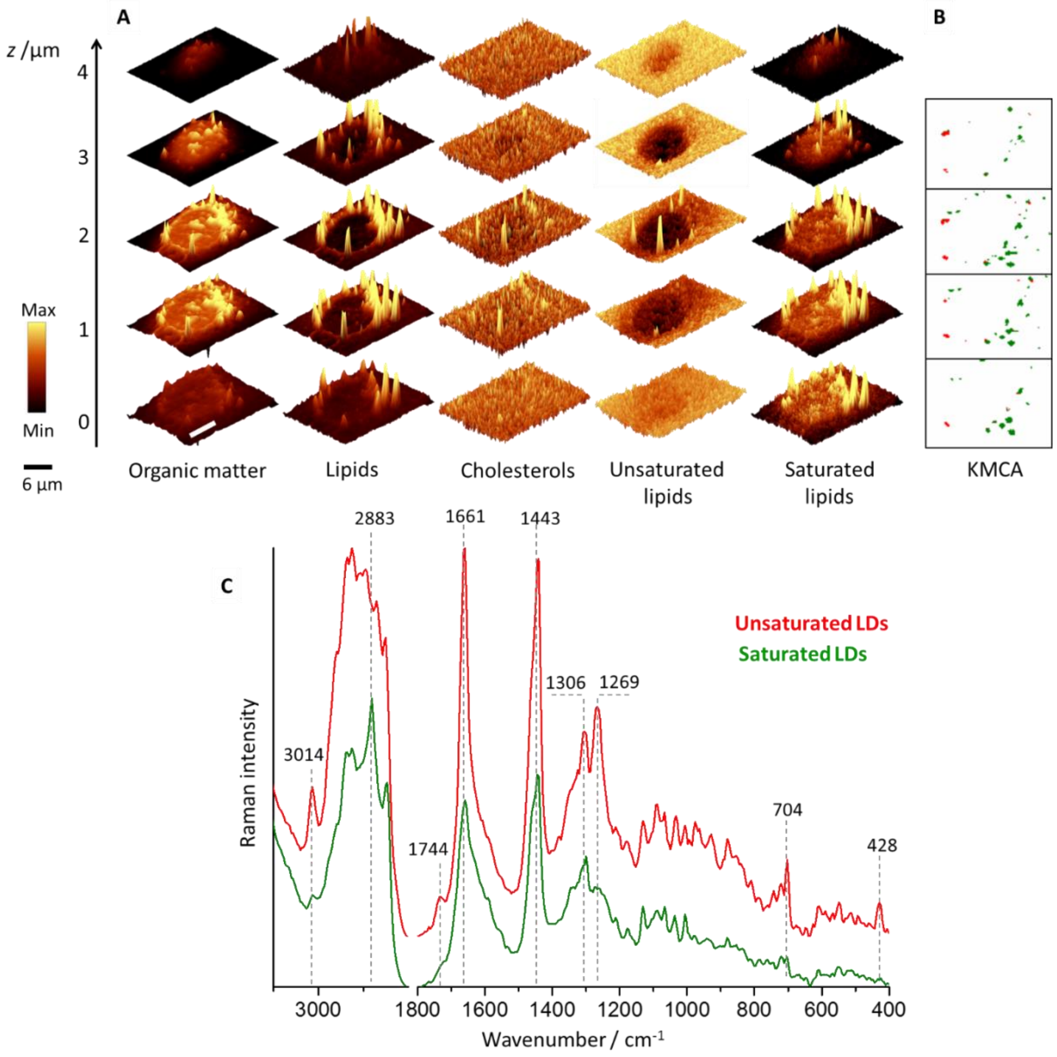

Figure S3. Confocal 3D imaging of a random LPS-stimulated HMEC-1 cell. Raman distribution image for representative 24 h LPS-stimulated (10 µg·mL^{-1}) cell (A) obtained from layers every 1 µm step in the z-direction by integration in the spectral region of 3030–2830 cm^{-1} (all organic matter), 2900–2830 cm^{-1}, 715–695 cm^{-1} (cholesterols), 3030–3000 cm^{-1} (unsaturated lipids) and 2900-2870 cm^{-1} (saturated lipids). Intensities of bands in all layers were normalized. The KMCA images (B) from the studied planes in which two classes were identified in colors: unsaturated LDs (red) and saturated LDS (green) with the corresponding averaged Raman spectra of classes (C). Spectra were normalized in the 1500–400 cm^{-1} spectral range and have been shifted vertically for clarity.

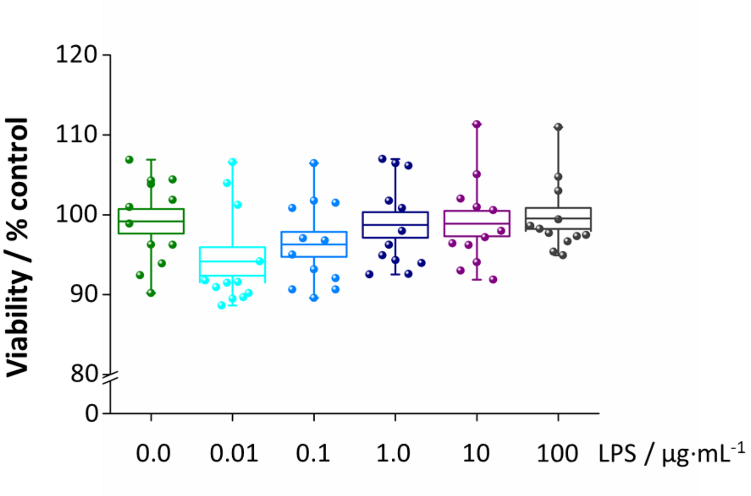

Figure S4. Changes in cell viability in HMEC-1 treated with different concentrations of LPS. The MTS assay was performed for cells maintained in FBS-containing medium supplemented with LPS for 24 h. Values given as mean ± SEM are shown in box plots: mean (horizontal line), SEM (box), minimal and maximal values (whiskers).
